# Traditional foods and 25(OH)D concentrations in a subarctic First Nations community

**DOI:** 10.3402/ijch.v75.31956

**Published:** 2016-09-22

**Authors:** Sudaba Mansuri, Alaa Badawi, Sheena Kayaniyil, David E. Cole, Stewart B. Harris, Mary Mamakeesick, Thomas Wolever, Joel Gittelsohn, Jonathon L. Maguire, Philip W. Connelly, Bernard Zinman, Anthony J. Hanley

**Affiliations:** 1 Department of Nutritional Sciences, University of Toronto, Toronto, ON, Canada; 2 Office of Biotechnology, Genomics and Population Health, Public Health Agency of Canada, Toronto, ON, Canada; 3 Sunnybrook Research Institute, Sunnybrook Health Sciences Centre, Toronto, ON, Canada; 4 Center for Studies in Family Medicine, Schulich School of Medicine and Dentistry, University of Western Ontario, London, ON, Canada; 5 Sandy Lake Health and Diabetes Project, Sandy Lake, ON, Canada; 6 Johns Hopkins Bloomberg School of Public Health, Johns Hopkins University, Baltimore, MD, USA; 7 Li Ka Shing Knowledge Institute of St. Michael's Hospital, Toronto, ON, Canada; 8 Lunenfeld-Tanenbaum Research Institute, Mount Sinai Hospital, Toronto, ON, Canada

**Keywords:** 25(OH)D, determinants, First Nations, traditional foods, traditional activities

## Abstract

**Background:**

Sub-optimal vitamin D status is common worldwide and the condition may be associated with increased risk for various chronic diseases. In particular, low vitamin D status is highly prevalent in indigenous communities in Canada, although limited data are available on the determinants of serum 25-hydroxyvitamin D (25(OH)D) concentrations in this population. The relationship between traditional food consumption and vitamin D status has not been well documented.

**Objective:**

To investigate the determinants of serum 25(OH)D status in a First Nations community in Ontario, Canada, with a focus on the role of traditional food consumption and activities.

**Methods:**

A cross-sectional analysis was conducted within the Sandy Lake Health and Diabetes Project (2003–2005). A total of 445 participants (>12 years of age) were assessed for serum 25(OH)D status, anthropometric and lifestyle variables, including traditional and non-traditional dietary practices and activities. Diet patterns were identified using factor analysis, and multivariate linear regression analysis was used to analyse the determinants of 25(OH)D concentrations.

**Results:**

Mean serum 25(OH)D concentrations were 22.1 nmol/L (16.9, 29.9 nmol/L) in men and 20.5 nmol/L (16.0, 27.3 nmol/L) in women. Multivariate determinants of higher serum 25(OH)D included higher consumption of traditional and healthier market foods, higher wild fish consumption, male gender, spring/summer season of blood collection and more frequent physical activity. Significant negative determinants included hours of TV/day, higher BMI and higher consumption of unhealthy market foods.

**Conclusions:**

Traditional food consumption contributed independently to higher 25(OH)D concentrations in a First Nations community with a high prevalence of sub-optimal vitamin D status.

Sub-optimal vitamin D status is an important public health problem due to the risk of adverse bone outcomes and the emerging possibility that it may be a risk factor for other chronic diseases such as cardiovascular disease, cancer and diabetes (1). Although sub-optimal vitamin D status is prevalent worldwide (2), specific groups have been documented to have notably high prevalence rates, including migrant populations in European countries (3,4), Middle Eastern populations (5–7), as well as indigenous populations in North America (8–10) and Australia (11).

Previous studies of the determinants of sub-optimal vitamin D status have identified factors such as low vitamin D intake (i.e. vitamin D containing foods and supplement use), high body mass index (BMI), winter season, reduced sun exposure and darker skin pigmentation (5,12–15). Although a high prevalence of vitamin D deficiency (serum 25(OH)D <50 nmol/L) (8,9,15–17) and sub-optimal vitamin D intake (16,18) has been reported in indigenous populations in Canada, relatively few studies have investigated the determinants of sub-optimal vitamin D status, which is of interest as these communities face many unique challenges including rapid nutrition transition (19,20), food insecurity (21) and living at northern latitudes (22). A recent study in the Eastern James Bay Cree found that vitamin D insufficiency was predicted by low fish and milk intake, obesity, younger age, spring blood collection and low vigorous physical activity (12).

Little is known regarding the importance of vitamin D status of traditional foods and activities, which are potentially good sources of vitamin D (i.e. wild fish and game, hunting/fishing and other outdoor activity), but which have declined markedly over time due to nutritional transition and the adverse impact of acculturation (18,20,23,24). The objective of this study, therefore, was to investigate the determinants of serum 25(OH)D status in an indigenous community in Ontario, Canada. We hypothesized that in addition to known correlates (age, gender, sun exposure, sunscreen use, supplement use, fortified foods) of vitamin D status, the consumption of traditional foods and participation in traditional activities would contribute independently to increased 25(OH)D status in this population.

## Materials and methods

The Sandy Lake Health and Diabetes Project (SLHDP) is a population-based study designed to determine the incidence of type 2 diabetes mellitus (T2DM) and associated risk factors in Sandy Lake, 53.1°N, 93.3°W, a remote indigenous community in northern Ontario, Canada (see Supplementary Fig. 1). The study has been approved by the Sandy Lake First Nation Band Council and the University of Toronto Research Ethics Board. The present analysis is a cross-sectional evaluation conducted using data from one cycle of the SLHDP, a community-based study of diabetes and associated risk factors. Between 2003 and 2005, 485 individuals participated in an evaluation of diabetes and its associated risk factors including obesity, impaired glucose tolerance and impaired fasting glucose (25). After excluding participants who did not have saved serum samples for 25(OH)D, a total of 445 participants (>12 years of age) were included in the present analysis.

Serum 25(OH)D was measured using the DiaSorin 25-OH Vitamin D TOTAL competitive chemiluminescence immunoassay on an automated LIAISON^®^ analyzer (Stillwater, MN). This assay has 100% specificity for both 25(OH)D_2_ and 25(OH)D_3_, with a detection limit of 10 nmol/L. The 25(OH)D TOTAL method has been validated against the DiaSorin radioimmunoassay (RIA) (r=0.92). This is a widely used method and was the first 25(OHD)D test approved for clinical diagnosis by the FDA (26). Since the time the aliquots were initially drawn and processed, they had been stored at −80°C for an average of 7 years until the time of assaying; under these conditions, 25(OH)D has been shown to be stable in serum or plasma (27,28). In order to confirm the observed 25(OH)D concentrations, the samples were re-run on a second LIAISON analyzer, and in addition a subsample was repeated on a Roche Modular system. In both cases, results were within 5% of the original values from the first LIAISON analyzer run. Further, Mount Sinai Services Laboratory (the laboratory that ran the assays) participated in the DEQAS program. Reports from the time period during which these assays were run indicate that results from the lab (DiaSorin LIAISON) had a bias of ±7.6% or lower from the method mean.

Parathyroid hormone (PTH) was measured using an electrochemiluminescence immunoassay on the Roche Modular E170 Analyzer (Laval, Quebec, Canada). It had a detection range from 0.127 to 530 pmol/L.

Height and weight were measured using an Accustat wall-mounted stadiometer (Genentech Inc., San Francisco, California) and a hospital balance beam scale (Health-o-Meter Inc., Bridgeview, Illinois), respectively. BMI was calculated as weight/height^2^ (kg/m^2^). Waist circumference was measured to the nearest 0.5 cm at the iliac crest; the hips were measured to the nearest 0.5 cm at the maximum extension of the buttocks. The percentage of body fat was estimated by bioelectrical impedance analysis using the Tanita TBF-201 Body Fat Analyzer (Tanita Corp., Tokyo, Japan) which has been validated in patients with T2DM (29). Systolic and diastolic blood pressure were determined at the appearance of first and fifth Korotkoff sounds, respectively. Each anthropometric and blood pressure measurement was performed twice, and the average was used in analyses.

A structured vitamin D questionnaire was adapted from Sahota et al. (30) to collect information on sun exposure and dietary sources of vitamin D-containing foods over the previous month. We used qualitative interviews and pilot testing to adapt this questionnaire to include traditional vitamin D-containing foods available in Sandy Lake. Commonly consumed traditional dishes that arose from the qualitative interviews and pilot testing included wildfish, such as Whitefish, Pickerel, Northern Pike, Goldeye, Burbot, Sturgeon, Trout and offal (“fried fish guts”). In the analysis, wild fish consumption was categorized as a dichotomous variable: greater than or equal to once per month, or less than once per month. All questionnaires were conducted in-person and were interviewer-administered.

A version of the Modifiable Activity Questionnaire (MAQ) (originally developed for the Pima Indian community) was used to collect information on physical activity, leisure and occupational activities, as well as sedentary activities including television watching (31). This questionnaire was adapted using qualitative research methods to include common physical activities practiced in Sandy Lake (29). Participant's physical activity was determined over the previous year, expressed as hours per week and was weighted by a crude estimate of the metabolic cost of each activity (MET). MET is the ratio of the working metabolic rate of an activity divided by the resting metabolic rate. Therefore, the level of physical activity was expressed as MET hours per week. The MAQ was an in-person interviewer-administered questionnaire.

The frequency of participation in traditional outdoor activities, including camping, fishing, trapping and hunting, was determined using a standardized questionnaire. These activities were combined into a single variable for the analysis and are expressed as times/year.

Dietary intake was determined using a 36-item food frequency questionnaire (FFQ). This instrument was developed using qualitative research methods and included both traditional and store-bought (market) foods. Participants recalled their usual diet over the previous 3 months (32).

Dietary intake data from this instrument was used to determine dietary patterns using factor analysis. Exploratory factor analysis was conducted, using the FACTOR procedure in SAS 9.4 (SAS Institute Inc. Cary, NC, USA), utilizing the “principal factors” (“method=prin”) and “priors=smc” options with a promax (oblique) rotation. The number of factors (or patterns) retained in the factor analysis was determined using a scree plot and interpretability criteria, in addition to a cut-point of 15% for the minimum common variation explained (33,34). A loading cut-point of ≥0.30 was used to interpret factor loadings of the FFQ items. Using a three-factor solution (described below), factor scores were calculated for each study participant by multiplying their frequency of intake of FFQ items by the loading for each of the food patterns. The factor scores for each pattern were then used as exposure variables in linear regression models.

### Statistical analysis

All analyses were conducted using SAS version 9.4 (SAS Institute, Cary, NC), and with the consideration of two-sided p<0.05 as statistically significant. Continuous variables were summarized as mean±standard deviation (SD) or median with interquartile range for variables with a skewed distribution. Categorical variables were reported as n (%). Chi-square tests were performed to test equality of proportions for categorical variables, and ANOVA tests were performed to determine the significance of differences of means across categories. Spearman correlation analyses were conducted to assess univariate associations between continuous variables.

Multiple linear regression analysis was used to analyse the multivariate determinants of serum 25(OH)D concentration, which was specified as the dependent variable in these models. Six nested multivariate models were specifically constructed to assess independent associations of previously documented determinants as well as traditional foods and activities on 25(OH)D status. Model 1 included age, sex, TV hours/day, season of 25(OH)D measurement, MET hours/week and BMI; model 2 included model 1 variables in addition to dietary patterns; model 3 included model 1 variables in addition to wild fish consumption, model 4 included model 1 variables in addition to wild fish consumption (excluding offal (fried fish guts)); model 5 included model 1 variables in addition to offal consumption, and model 6 included model 1 variables in addition to participation in traditional activities.

Sensitivity analyses were conducted to assess the impact of other adiposity measures, specifically body fat percentage, waist circumference (measured based on Health Canada values (35)) and waist to hip ratio, as alternate variables in the multiple linear regression analysis. All adiposity measures were used as continuous variables in multiple linear regression analysis. Another set of sensitivity analyses were conducted to assess the impact on models 1–6 of excluding participants with T2DM, who are known to have lower 25(OH)D concentrations.

In order to address the possible correlations between variables that could lead to multicollinearity, we used the variance inflation factor (VIF) option in PROC REG (regression procedure) in SAS. A VIF of >2 indicates the presence of multicollinearity. Our findings suggested that there was no multicollinearity present in the regression analysis as all VIFs were <2 (data not shown).

## Results

A total of 445 participants from the SLHDP project were included in this study, with blood collection in fall (n=80), winter (n=154), spring (n=149) and summer (n=62). The median serum 25(OH)D concentration was 23.2 (16.1, 27.9) nmol/L, in fall 23.0 (16.6, 30.0) nmol/L, winter 18.1 (14.2, 22.9) nmol/L, spring 23.0 (17.5, 29.2) nmol/L and summer 25.8 (19.8, 34.9) nmol/L. According to the Institute of Medicine's (IOM) cut-offs for 25(OH)D (36), 98.4% of participants had 25(OH)D levels of <50 nmol/L and 80.9% of participants had 25(OH)D levels of <30 nmol/L. Mean serum 25(OH)D concentrations were 22.1 nmol/L (16.9, 29.9) in men and 20.5 nmol/L (16.0, 27.3) in women, and 19.7 nmol/L (15.6, 26.7) in participants ≤34 years and 22.8 nmol/L (17.6, 29.9) in participants >34 years of age. Lower serum 25(OH)D was found among smokers, non-sunscreen users, obese participants, and those who had their blood collected in the winter months (all p<0.05) (Table I).

**Table I:** Between category differences in mean 25(OH)D (nmol/L) according to demographic and behavioural factors (n=445)

Variables	*n*	25(OH)D	*p*
Age (years)			
≤34	231	19.7 (15.6, 26.7)	0.001
>34	214	22.8 (17.6, 29.9)	
Gender			
Male	181	22.1 (16.9, 29.9)	0.04
Female	264	20.5 (16.0, 27.3)	
Current smoker			
Yes	288	20.5 (15.9, 27.7)	0.02
No	109	23.1 (17.6, 29.2)	
Supplement use			
Yes	74	21.4 (16.4, 27.7)	0.71
No	319	21.2 (15.8, 28.1)	
Sun exposure			
Days outside/week			
<7	109	23.6 (18.7, 29.5)	0.17
7	41	27.2 (19.8, 34.2)	
Limbs covered			
Yes	45	23.8 (17.5, 30.5)	0.85
No	22	22.6 (17.5, 31.1)	
Partial	87	24.8 (19.5, 30.0)	
Sunscreen use			
Yes	32	28.3 (24.2, 32.3)	0.03
No	120	23.3 (17.9, 29.5)	
BMI (kg/m^2^)			
Healthy weight (<25)	102	20.4 (15.3, 29.8)	0.007
Overweight (≥25–29.9)	135	23.6 (17.8, 29.5)	
Obese (≥30)	197	19.8 (16.1, 25.8)	
Season of blood collection			
Fall	80	23.0 (16.6, 30.0)	<0.001
Winter	154	18.1 (14.2, 22.9)	
Spring	149	23.0 (17.5, 29.2)	
Summer	62	25.8 (19.8, 34.9)	

Continuous variables are shown as means±SD for normally distributed variables, or median (25 and 75% interquartiles) for non-normally distributed variables and no. (%) for categorical variables. Fall: October–December; Winter: January–March; Spring: April–June; Summer: July–September. Age=median cut-off.

The three dietary patterns retained from factor analysis included a balanced market foods pattern, a Western diet-market foods pattern and a traditional foods pattern (Supplementary Table I). The balanced market foods pattern was characterized by cold cereals, whole wheat bread, potatoes, peas, corn, carrots, other vegetables, fresh fruit and milk. The Western diet-market foods pattern was characterized by beef/steak/hamburger, pork chops/bacon, canned luncheon meat, eggs, white bread, chips/French fries, canned fruit, pop/soda, cookies/cake/pastries, chocolate/candy, fried food from fast food restaurants. Finally, the traditional foods pattern was characterized by fish, moose meat, duck or goose, rabbit, Indian medicine or tea, home-made soup and wild berries (Supplementary Table I). We assessed the association of 25(OH)D concentration with these three patterns and found a significant inverse correlation with the Western diet-market foods pattern (r=−0.12, p<0.05) and significant positive correlations with the balanced market foods pattern (r=0.13, p<0.01) and the traditional foods pattern (r=0.12, p<0.05).

In univariate analysis, there were significant inverse correlations of 25(OH)D with PTH, BMI, percent body fat percentage, hip circumference and TV watching (Table II). There were also significant positive correlations between 25(OH)D and age, total physical activity, fishing, camping, hunting and traditional activities (all p<0.05) (Table II).

**Table II:** Spearman correlations of serum 25(OH)D with continuous variables

Variables	25(OH)D r
PTH (pmol/L)	−0.33***
Age (years)	0.23***
Body mass index (kg/m^2^)	−0.11*
% Body fat	−0.11*
Waist circumference, cm	−0.08
Waist to hip ratio	0.04
Hips, cm	−0.10*
Total physical activity (MET hrs/week)	0.14**
Watching TV (hours/day)	−0.17***
Balanced market foods pattern	0.13**
Western diet-market foods pattern	−0.12*
Traditional foods pattern	0.12*
Fishing (times/year)	0.11*
Camping (times/year)	0.11*
Trapping (times/year)	0.078
Hunting (times/year)	0.13**
Traditional activity (times/year)	0.12*

r representes Spearman correlations

* p<0.05

** p<0.01

*** p<0.001.

Traditional activity includes: camping + fishing + trapping + hunting.

**Fig. 1:**
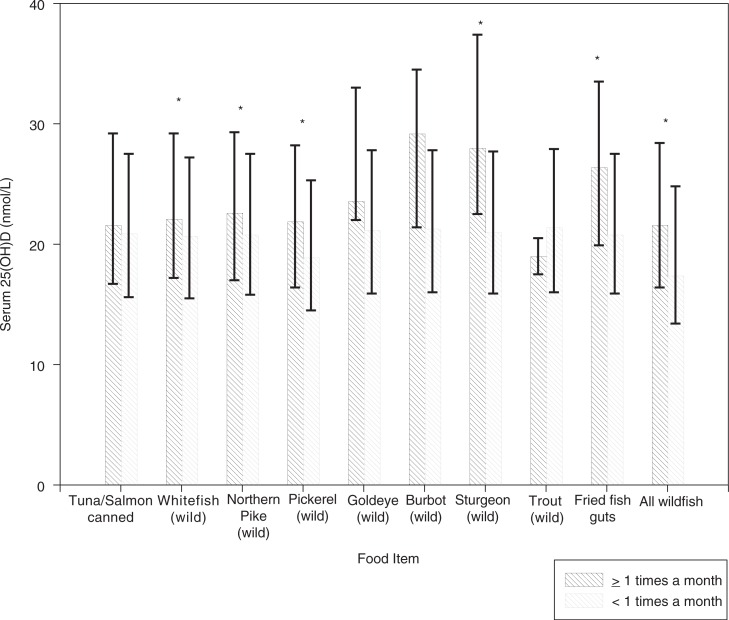
Serum 25(OH)D concentrations according to consumption of different types of fish. Error bars represent interquartile range, *p<0.05.

Participants who consumed whitefish, Northern pike, pickerel, sturgeon, fried fish guts and all wild fish ≥1 time per month compared to participants who consumed these food items less than once per month had significantly higher 25(OH)D concentrations (all <0.01) (Figure 1).

Multiple linear regression analysis was conducted and six models were used to assess the determinants of 25(OH)D concentration, as described in Methods. Model 1 indicated significant positive associations of 25(OH)D with age, spring/summer season and physical activity; and negative associations of 25(OH)D with TV hours watched/day and BMI (Table III). Model 2 showed significant positive associations between 25(OH)D with the balanced market foods pattern and the traditional market foods pattern, and a negative association with the Western-diet foods pattern (Table III). Wild fish consumption including or excluding fried fish guts (models 3 and 4 respectively) was significantly associated with 25(OH)D concentrations. In models 2, 3 and 4, associations with other variables from model 1 remained similar in terms of direction and significance (Table III). Neither fried fish guts consumption alone nor traditional activities were significantly associated with 25(OH)D after multivariate adjustment (Table III, models 5 and 6).

**Table III:** Multivariate linear regression analysis of 25(OH)D with traditional and non-traditional variables

Variables	Model 1 β (Standard errors)	Model 2 β (Standard errors)	Model 3 β (Standard errors)	Model 4 β (Standard errors)	Model 5 β (Standard errors)	Model 6 β (Standard errors)
**Core variables:**						
Age	0.14 (0.03)***	0.04 (0.04)	0.16 (0.03)***	0.17 (0.04)***	0.15 (0.04)***	0.14 (0.04)***
Sex	1.24 (0.96)	2.25 (1.06)*	1.57 (0.95)	1.57 (0.95)	1.91 (0.97)	1.08 (0.97)
TV hours/day	−0.70 (0.21)**	−0.70 (0.22)**	−0.65 (0.21)**	−0.70 (0.21)**	−0.70 (0.21)**	−0.70 (0.21)**
Season	4.54 (0.84)***	4.81 (0.90)***	4.50 (0.84)***	4.45 (0.84)***	4.30 (0.84)***	4.47 (0.85)***
MET hours/week	0.01 (0.004)***	0.01 (0.005)*	0.01 (0.004)***	0.01 (0.004)***	0.02 (0.004)***	0.01 (0.005)**
BMI	−0.24 (0.07)**	−0.27 (0.08)**	−0.24 (0.07)***	−0.21 (0.07)**	−0.20 (0.07)**	−0.24 (0.07)***
Balanced market foods pattern		1.21 (0.53)*				
Western-diet foods pattern		−1.72 (0.61)**				
Traditional foods pattern		1.66 (0.57)**				
Wild fish consumption (times/month)			2.81 (1.22)*			
Wild fish consumption^a^				2.77 (1.21)*		
Fried fish guts (times/month)					2.49 (1.39)	
Traditional activities (times/year)						0.01 (0.01)

* p<0.05

** p<0.01

*** p<0.001

a Wildfish consumption excluding fried fish guts consumption; Data are beta estimates (standard errors); sex=1 (male), 2=(female); season=1 (spring/summer), season=2 (fall/winter); BMI is used as a continuous variable.

In sensitivity analyses which assessed the impact of including different adiposity measures in our models, specifically body fat percentage, waist circumference and waist to hip ratio in comparison to BMI, findings were similar to those for BMI, except for waist to hip ratio which was not associated with 25(OH)D (Supplementary Table II). In another set of sensitivity analyses, in which n=122 participants with T2DM were excluded, the majority of the associations with 25(OH)D remained similar, with the exception of the balanced market foods diet pattern score which was no longer associated with 25(OH)D (Supplementary Table III).

## Discussion

In the present study, we investigated determinants of 25(OH)D concentrations in a First Nations population in northern Ontario, Canada, with sub-optimal vitamin D status. A cross-sectional analysis using data from the SLHDP showed that 25(OH)D was inversely associated with TV hours watched/day, higher BMI and a Western dietary pattern, and positively associated with wild fish consumption, spring/summer season as well as balanced market and traditional diet patterns.

The median concentration of 25(OH)D in this population was markedly lower than reported concentrations in the general population of Canadians (37). According to a 2011 Statistics Canada report, using data from the Canadian Health Measures Survey, the average mean concentration of 25(OH)D for females was 67 nmol/L while the average for males was 61 nmol/L (37). Previous studies in indigenous communities in Canada have reported a range of 25(OH)D concentrations, including two studies reporting similar levels to the present study (9,16). Young children in a northern Manitoba community were found to have a serum 25(OH)D concentration of 26.2 nmol/L while mothers had a serum 25(OH)D concentration of 19.8 nmol/L (9). Another cross-sectional study by El Hayek et al. (16) reported a median serum 25(OH)D concentration of 37.7 (21.4–52.0) nmol/L in the winter. In addition, similarly low 25(OH)D concentrations have been reported in other populations, including those in the Middle East and immigrant populations of Europe. Vitamin D deficiency was prevalent in medical students from Saudi Arabia, where 75.2% had 25(OH)D levels <30 nmol/L (5). Also, the prevalence of vitamin D deficiency (serum 25(OH)D <50 nmol) was 80% in premenopausal and 68% postmenopausal Saudi female outpatients (7). A similar prevalence of deficiency was noted in immigrant populations of European countries. For instance, severely low 25(OH)D status (serum 25(OH)D <30 nmol/L) was evident in a population of Pakistani immigrants in Denmark (3). Serum 25(OH)D concentration of recent immigrants from Africa and Asia in Norway showed highest prevalence of deficiency among immigrants from the Middle East, followed by those from South Sahara Africa and South Asia (4) (see Supplementary Fig. 2).

In the current study, our findings of negative associations of 25(OH)D with BMI and TV watching, and positive associations with spring/summer season and physical activity are essentially consistent with what has been reported previously (13,14). Among participants in our study, women had lower 25(OH)D concentrations compared to men. Although this gender difference is not always observed (37), it has been documented in other indigenous groups in Canada (8,12). These findings are important as vitamin D insufficiency has been associated with an increased risk of gestational diabetes and may impact the musculoskeletal health of offspring (38,39). Additionally, our finding of a positive association of age with 25(OH)D is not generally consistent with published literature (40). Although previous results on the effect of age on serum 25(OH)D are mixed, our finding is consistent with those for other indigenous groups in Canada (12,41). These findings could be partly attributed to the more frequent engagement in traditional activities (i.e. hunting, fishing, trapping), and the consumption of traditional foods by older people in the community.

Very few previous studies have investigated determinants of low vitamin D status among indigenous populations in Canada. A study by Riverin et al. (12) found that low fish and milk intake, obesity, younger age, spring blood collection and low vigorous physical activity were all associated with sub-optimal vitamin D status in James Bay Cree. We documented similar associations, and taken together, the data from these studies highlight the importance of improving environments for physical activity and access to healthy market foods in indigenous communities in Canada. A previous landmark study demonstrated that oily fish, such as wild salmon is an excellent source of vitamin D (42); however, this was not apparent in our study. This finding can be explained due to the fact that wild salmon is not available in this community. Only canned salmon is available, and it is not consumed commonly. Our study extends this literature by highlighting the importance of aspects of traditional lifestyles with regard to vitamin D levels in this population. We measured specific factors such as participation in traditional activities (camping, fishing, hunting, trapping) and consumption of traditional foods (wild fish, fried fish guts) to determine their association with vitamin D status in the First Nations population. Our observations indicated that consuming traditional foods was independently associated with better vitamin D status. These findings may be explained in part through engaging in behaviours which increase in sun exposure and physical activity, and consuming foods with higher vitamin D content (12,40). Further, the FFQ queried the previous 3 months only, which would have misclassified dietary intake to a degree, resulting in conservative estimates of the impact of diet patterns on 25(OH)D concentrations.

The strengths of this study include the use of specifically tailored questionnaires for this population such as the FFQ, the MAQ, and the vitamin D questionnaire. This study also contributes important information on the role of traditional factors as determinants of vitamin D status in this population. This study, does, however, have a number of limitations that need to be considered when interpreting the results, including its cross-sectional design which does not permit the evaluation of the temporal association of 25(OH)D with the aforementioned determinants. In addition, the FFQ likely resulted in misclassification of dietary intake due to the limited number of items on the instrument and imperfect recall of past diet among the study participants. Further, as this instrument is a short (36 item) non-quantitative FFQ, originally designed to examine diet patterns in this community (32,43), the instrument does not contain information on portion sizes. Therefore, we do not have macro- or micro-nutrient information for the participants from this FFQ. Additionally, the FFQ did not include questions on seasonality of consumption of individual foods; as a result, the relationships of seasonal fluctuations in food consumption in relation to 25(OH)D status were not documented. Finally, data for each participant were collected once, which did not allow us to evaluate intra-individual fluctuations in 25(OH)D status by season, or the determinants of these fluctuations.

## Conclusion

Our results suggest that the consumption of traditional foods (wild fish, in particular) contributes independently to better vitamin D status in a northern First Nations community with a high prevalence of sub-optimal vitamin D status. Understanding the determinants of 25(OH)D levels may assist in identifying those at risk of low vitamin D status and increasing awareness about foods and practices that contribute to healthy vitamin D status. These findings have been communicated to community members and stakeholders using a variety of approaches, and the information is being incorporated into ongoing community-based prevention programming (44).

## Supplementary Material

Traditional foods and 25(OH)D concentrations in a subarctic First Nations community

Traditional foods and 25(OH)D concentrations in a subarctic First Nations community
