# Supplementary material for: Traditional foods and 25(OH)D concentrations in a subarctic First Nations community
Source: Int J Circumpolar Health. 2016 Sep 22;75:10.3402/ijch.v75.31956. doi: 10.3402/ijch.v75.31956 (PMC5035507; doi:10.3402/ijch.v75.31956)
Supplement: Traditional foods and 25(OH)D concentrations in a subarctic First Nations community [file IJCH-75-31956-s001.docx]

**SUPPLEMENTARY TABLES**

**Figure S1.**


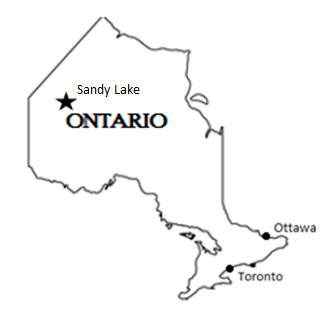


**Figure S2. Concentration of 25(OH)D by study population/location**

| **Author (year)** | **Study Population/Location** | **Vitamin D concentration** |
| --- | --- | --- |
| Lebrun et al 1993 | Young children in a Northern Manitoba community | Children: 26.2 nmol/L;  Mothers: 19.8 nmol/L |
| El Hayek et al 2010 | Inuit preschoolers | summer and winter; 48.3 (32.8–71.3) and 37.7 (21.4–52.0) nmol/L respectively |
| Andersen et al 2008 | Pakistani immigrants in Denmark | The median concentration of serum 25(OH)D was 10.9, 12.0 and 20.7 nmol/l for girls, women and men, respectively. |
| BinSaeed et al 2015 | Medical students from Saudi Arabia | 75.2% had 25(OH)D levels < 30 nmol/L |
| Kanan et al 2013 | Saudi female outpatients | Vitamin D deficiency (serum 25(OH)D < 50 nmol/L) was 80% in premenopausal and 68% postmenopausal women |
