# Supplementary material for: Traditional foods and 25(OH)D concentrations in a subarctic First Nations community
Source: Int J Circumpolar Health. 2016 Sep 22;75:10.3402/ijch.v75.31956. doi: 10.3402/ijch.v75.31956 (PMC5035507; doi:10.3402/ijch.v75.31956)
Supplement: Traditional foods and 25(OH)D concentrations in a subarctic First Nations community [file IJCH-75-31956-s002.pdf]

SUPPLEMENTARY TABLES

Table S1. Factor Loadings for each food as listed on the 36-item FFQ

FFQ Items	Balanced Market Foods	Western diet-market foods	Traditional Foods
Fish	1	-17	48*
Moosemeat	3	3	40*
Beef /steak / hamburger	3	44*	-14
Pork chops or bacon	12	44*	0
Chicken	13	33*	3
Duck or goose	-4	0	60*
Rabbit	-2	9	59*
Klik or spork	-12	34*	28
Eggs	3	40*	-7
Butter/lard	2	27	10
Margarine	14	9	-4
Cold cereals	34*	31*	-8
Hot cereals	30*	-6	17
Beans	12	7	25
White bread	-1	35*	1
Whole wheat bread	32*	-2	6
Bannock	5	9	28
Macaroni or other pasta	14	22	5
Indian medicine or Indian tea	-7	-6	39*
Home-made soup	23	6	31*
Chips or French fries	0	65*	-4
Other potatoes	33*	18	2
Peas	64*	-6	-2
Corn	61*	5	-2
Carrots	67*	-8	2
Other vegetables	64*	-9	3
Wild berries	22	0	31*
Fresh fruit from the store	37*	3	11
Canned fruit	13	34*	15
Milk	34*	15	-12
Carnation milk	-4	8	17
Pop/soda	-5	42*	-9
Tea/coffee	-12	-2	8
Cookies/cake/Pastries	-3	36*	17
Chocolate/ candy or bars	-7	39*	17
Fried food from fast food restaurants	-4	47*	2

Three-factor factor analysis solution with orthogonal rotation; Eigenvalues (loadings) shown as eigenvalue*100 for simplicity; Loadings >= 30 *

Table S2. Multivariate linear regression analysis of 25(OH)D with core variables: sensitivity analysis of adiposity measures: body fat percentage, waist circumference and waist to hip ratio

**Variables**	**Model 1**	**Model 2**	**Model 3**	**Model 4**
**Core Variables:**				
Age	0.14 (0.03)†	0.13 (0.04) †	0.16 (0.04) †	0.14 (0.04) †
Sex	1.24 (0.96)	0.93 (1.28)	-1.88 (0.95)*	-2.49 (1.06)*
TV hours/day	-0.70 (0.21)‡	-0.79 (0.22) †	-0.69 (0.21) ‡	-0.74 (0.21) †
Season	4.54 (0.84)†	-4.71 (0.90) †	-4.61 (0.84) †	-4.57 (0.85) †
MET hours/week	0.01 (0.004)†	0.01 (0.005) †	0.01 (0.004)†	0.01 (0.004) †
BMI	-0.24 (0.07)‡			
Body fat percentage		-9.78 (3.67) ‡		
Waist circumference			-0.12 (0.03) †	
Waist to hip ratio				-10.33 (6.45)

* $p < 0.05$, ‡ $p < 0.01$, † $p < 0.001$

Data are beta estimates (standard errors)

Table S3. Multiple linear regression analysis of 25(OH)D: sensitivity analysis excluding participants with T2DM

Variables	Model 1<br>β (Standard Error)	Model 2<br>β (Standard Error)	Model 3<br>β (Standard Error)	Model 4<br>β (Standard Error)	Model 5<br>β (Standard Error)	Model 6<br>β (Standard Error)
**Core Variables:**						
Age	0.16 (0.03)†	0.06 (0.05)	0.14 (0.04)†	0.14 (0.04)†	0.13 (0.04)†	0.15 (0.04)†
Sex	1.68 (1.04)	2.59 (1.14)*	1.63 (1.04)	1.62 (1.04)	1.91 (1.05)	1.40 (1.08)
TV hours/day	-0.66 (0.21\3)‡	-0.64 (0.24)‡	-0.61 (0.23)‡	-0.60 (0.23)‡	-0.66 (0.23)‡	-0.63 (0.23)‡
Season	3.93 (0.93)†	4.34 (0.98)†	4.07 (0.92)†	4.09 (0.92)†	3.82 (0.92)†	3.85(0.85)†
MET hours/week	0.02 (0.005)‡	0.01 (0.005)*	0.01 (0.005)†	0.01 (0.005)‡	0.02 (0.005)†	0.01 (0.005)‡
BMI	-0.19 (0.08)*	-0.20 (0.08)*	-0.19 (0.08)*	-0.19 (0.08)*	-0.17 (0.07)*	-0.19 (0.08)*
Balanced Market Foods pattern		0.67 (0.53)				
Western-Diet Foods pattern		-1.32 (0.61)*				
Traditional Foods pattern		1.71 (0.57)‡				
Wild Fish consumption (times/month)			2.80 (1.26)*			
Wild Fish consumption**				2.78 (1.25)*		
Fried fish guts (times/month)					3.18 (1.39)	
Traditional activities (times/year)						0.01 (0.01)

* p <0.05 , ‡ p <0.01, † p < 0.001; **Wildfish consumption excluding fried fish guts consumption ; Data are beta estimates (standard errors); sex=1 (male), 2= (female); season=1 (spring/summer), season=2 (fall/winter); * p <0.05 , ‡ p <0.01, † p < 0.001
